# Gross tumor volume of adenocarcinoma of esophagogastric junction corresponding to cT and cN stages measured with computed tomography to quantitatively determine resectabiliy: A case control study

**DOI:** 10.3389/fonc.2022.1038135

**Published:** 2022-11-17

**Authors:** Ke-ying Li, Jing Ou, Hai-ying Zhou, Zi-yi Yu, Dan Gao, Xin-yi You, Xiao-ming Zhang, Rui Li, Tian-wu Chen

**Affiliations:** Medical Imaging Key Laboratory of Sichuan Province, and Department of Radiology, Affiliated Hospital of North Sichuan Medical College, Nanchong, Sichuan, China

**Keywords:** esophagogastric junction, adenocarcinoma, tomography, X-ray computed, surgery, tumor burden

## Abstract

**Purpose:**

To determine whether gross tumor volume (GTV) of adenocarcinoma of esophagogastric junction (AEG) corresponding to cT and cN stages measured on CT could help quantitatively determine resectability.

**Materials and methods:**

343 consecutive patients with AEG, including 279 and 64 randomly enrolled in training cohort (TC) and validation cohort (VC), respectively, underwent preoperative contrast-enhanced CT. Univariate and multivariate analyses for TC were performed to determine factors associated with resectability. Receiver operating characteristic (ROC) analyses were to determine if GTV corresponding to cT and cN stages could help determine resectability. For VC, Cohen’s Kappa tests were to assess performances of the ROC models.

**Results:**

cT stage, cN stage and GTV were independently associated with resectability of AEG with odds ratios of 4.715, 4.534 and 1.107, respectively. For differentiating resectable and unresectable AEG, ROC analyses showed that cutoff GTV of 32.77 cm^3^ in stage cT_1-4_N_0-3_ with an area under the ROC curve (AUC) of 0.901. Particularly, cutoffs of 27.67 and 32.77 cm^3^ in stages cT_3_ and cT_4_ obtained AUC values of 0.860 and 0.890, respectively; and cutoffs of 27.09, 33.32 and 37.39 cm^3^ in stages cN_1_, cN_2_ and cN_3_ obtained AUC values of 0.852, 0.821 and 0.902, respectively. In VC, Cohen’s Kappa tests verified that the ROC models had good performance in distinguishing between resectable and unresectable AEG (all Cohen’s K values > 0.72).

**Conclusions:**

GTV, cT and cN stages could be independent determinants of resectability of AEG. And GTV corresponding to cT and cN stages can help quantitatively determine resectability.

## Introduction

Adenocarcinoma of the esophagogastric junction (AEG) is one of the most common cancers in the world, with an estimated 1 million deaths each year (1–3). In 1998, Siewert classified AEG into three types, including type I with the tumor center 1–5 cm above the esophagogastric junction, type II with the tumor center located between 1 cm above and 2 cm below the junction, and type III with the tumor center 2–5 cm below the junction. This classification has been adopted by the International Gastric Cancer Society and by the International Society for Disease of the Esophagus, and helped clarify that different approaches are needed for the different types of cancer (2, 4).

AEG usually adopts multimodal treatments, but the main treatment method is surgical resection (5). However, not all patients can benefit from surgery. The patients with locoregionally advanced or distant metastasis cannot undergo surgery and can only receive chemotherapy and/or radiotherapy (6). It is reasonable to consider that if the identifier of the unresectable tumor can be reliable, unnecessary or other ineffective surgery can be avoided (7, 8). Like other malignant diseases, the choice of the most appropriate treatment significantly affects the prognosis of patients with AEG. Therefore, determining the resectability of AEG is crucial for treatment decision-making (6). Accurate preoperative clinical staging plays an important role in determining the treatment strategy of patients. The American Joint Commission on Cancer (AJCC)/the Union for International Cancer Control (UICC) recommended that computed tomography (CT) of the chest and abdomen be used as an important method for staging clinical tumor lymph node metastasis (TNM) of advanced upper gastrointestinal tumors (9). Because previous studies have shown that gross tumor volume (GTV) of AEG is related to regional lymph node metastasis (10, 11), we speculate that the increase of tumor volume may be associated to local regional advanced or distant metastasis, which could ultimately impact on the determination of the feasibility of the tumor resectabiliy. However, to our knowledge, there are no studies to report if GTV of AEG could help determine the resectability. The objective of this study was to assess the factors associated with resectability of AEG, and feasibility of GTV measured with CT as one independent determinant corresponding to the other independent factors such as cT and cN stages to quantitatively determine the resectability.

## Materials and methods

### Patients

This retrospective study was approved by the institutional ethics committee of our hospital (Approval No. 2021ER020-1), and written informed consent was obtained from each participant before the study.

From October 2017 to November 2021, patients with AEG proved by endoscopic biopsy were enrolled into our study according to the following inclusion criteria: (a) the patient did not receive any tumor-related treatment (eg, radiation therapy and/or chemotherapy) before undergoing enhanced CT scanning, (b) the quality of CT was sufficient, and (c) the AEG lesion was regarded unresectable and resectable according to the NCCN guidelines based on CT findings (12, 13). The exclusion criteria were as follows: (a) patients had other malignant tumor history (n = 5); (b) because the patients with resectable AEG could not tolerate surgery or anesthesia, they did not receive surgical treatment but chemoradiotherapy (n = 3), or (c) the stomach showed poor filling on CT (n = 6). Finally, 343 patients were enrolled in this study, and the research subjects included 221 patients with resectable AEG, and 122 with unresectable tumors. Among patients with resectable AEG, 197 patients with primary resectable tumors did not receive neoadjuvant treatment but surgery, and the remained 24 patients received neoadjuvant treatment after CT and before surgical treatment. The tumors receiving neoadjuvant treatment shrank to be resectable after therapy, and these patients subsequently underwent successful surgery. All the 343 patients including the 24 cases achieving surgical resection after neoadjuvant chemotherapy were randomly divided into the training cohort (TC, n = 279) and a validation cohort (VC, n = 64).

### Definition of surgical resectability

All patients underwent enhanced CT scans of the thorax and upper abdomen. The resectable AEG lesions were clinically staged according to the AJCC staging system of AEG (10), and was confirmed by the postoperative histopathology. However, unresectable tumors were staged according to the radiology standard in the AJCC staging system (14, 15). On CT, the AEG presenting as only wall thickening was staged as <T_2_. Infiltration of periesophageal tissue was divided into stage T_3_ and was identified by high-density grounding of fat around the esophagus. Invasion of adjacent organs was staged as T_4_, and it is confirmed by the invasion of adjacent organs and the loss of intermediate fat layer. The diagnostic criteria of lymph node metastasis on CT was defined as lymph node enlargement with a short axis of more than 1 cm (16). According to the National Comprehensive Cancer Network Clinical Practice guidelines (NCCN Guidelines) in oncology (12, 13), we divided all patients into resectable and unresectable groups. In addition, it is mentioned that the treatment for AEG of Siewert types I and II has been described as esophageal cancer in the NCCN Guidelines. Siewert type III lesions are considered gastric cancers, and thus the NCCN Guidelines for gastric cancer should be followed in our study. The criteria of resectable AEG of Siewert types I/II were as follows: (a) T_1a_ tumors, defined as tumors involving the mucosa but not invading the submucosa, (b) tumors in the submucosa (T_1b_) or deeper, (c) T_1-3_ tumors were resectable even with regional nodal metastases, although bulky; and (d) T_4a_ tumors with involvement of pericardium, pleura, or diaphragm. The criteria of unresectable tumor of Siewert types I/II were as follows: (a) cT_4b_ tumors with involvement of the heart, great vessels, trachea, or adjacent organs including liver, pancreas, lung and spleen; (b) most patients with multi-station, bulky lymphadenopathy; (c) patients with supraclavicular lymph node involvement; and (d) patients with distant (including nonregional lymph nodes) metastases (stage IV). The criteria of unresectable AEG of Siewert type III were the tumors with locoregionally advanced and distant metastasis or peritoneal seeding (including positive peritoneal cytology). Exactly, disease infiltration of the root of the mesentery or para-aortic lymph node highly suspicious on imaging or confirmed by biopsy and invasion or encasement of major vascular structures (excluding the splenic vessels) were included in locoregionally advanced. If the AEG of Siewert type III was not regarded as unresectable, the cancer was considered resectable. According to the above staging criteria, the clinical characteristics, Siewert classification, cT stage, cN stage, vascular invasion and GTV are shown in **Table 1**. The detailed statuses of unresectable AEG such as distant metastasis in TC and VC are illustrated in **Table 2**. In addition, the clinical data including GTV on CT in the cases achieving surgical resection after neoadjuvant chemotherapy were obtained after this chemotherapy and before the surgical treatment.

**Table 1:** Demographic and clinical information of all enrolled patients.

Variable	Training cohort	Validation cohort
No. of patients with AEG (resectable: unresectable)	279 (184:95)	64 (37:27)
Sex, male: female	205:74	43:21
Age, median (range) in year	67 (33–85)	67 (38-82)
Siewert classification		
I	4	1
II	179	38
III	96	25
T stage		
cT_1_	8	1
cT_2_	23	6
cT_3_	102	21
cT_4a_	140	35
cT_4b_	6	1
N stage		
cN_0_	35	5
cN_1_	63	17
cN_2_	111	23
cN_3_	70	19
Vascular invasion, yes:no	97:182	18:46
GTV, mean ± SD (cm^3^)	36.58 ± 26.61	40.15 ± 45.52

GTV, gross tumor volume; and SD, standard deviation.

**Table 2:** The detailed patterns of enrolled cases with unresectable adenocarcinoma of esophagogastric junction.

Variable	Training cohort (n = 95)	Validation cohort (n = 27)
Siewert classification (I/II: III)	51:44	17:10
Patterns of Siewert type I/II		
Tumor stage cT4b	3 (5.9)	1 (5.9)
Multi-station and bulky lymphadenopathy	12 (23.5)	6 (35.3)
Supraclavicular lymph node involvement	4 (7.8)	1 (5.9)
Distant metastasis	13 (25.5)	2 (11.8)
Two or more kinds of above patterns	19 (37.3)	7 (41.1)
Patterns of Siewert type III		
Locoregionally advanced	17 (38.6)	3 (30.0)
Distant metastasis	10 (22.8)	4 (40.0)
Two kinds of above situations	17 (38.6)	3 (30.0)

The numbers in the parentheses are percentages.

### Contrast-enhanced CT scans

The CT data were obtained with two 64-section multidetector computed tomography (MDCT) scanners (LightSpeed VCT, GE Medical systems, USA). Before CT data acquisition, 800–1000 ml water was orally taken as negative contrast medium. All examinations were performed in the supine position. After routine unenhanced CT scanning, a total of 70–100 ml of contrast agent (Omnipaque, Iohexol, GE Healthcare, USA) calculated according to the proportion of 1.5 ml/kg body weight was customized at the rate of 3.0 ml/s through a 20-G needle with a pump injector (Vistron CT injection system, Medrad, USA) through a cubital vein, and subsequently 20 ml of saline was rinsed. The enhanced CT data of the arterial phase and portal venous phase were obtained 25 and 65 seconds after the injection of contrast agent, respectively. The scanning parameters are as follows: peak voltage of 120 kV, tube current of 200 mA (automatic exposure control), rotation time of 0.5 s, collimation of 64 × 0.6 mm, pitch of 0.9, matrix of 512 × 512 mm, and slice thickness of 5 mm. The coverage of CT scans were from the apex of the lungs though the liver to the middle of the right kidney in the arterial phase and from the right diaphragmatic dome to the middle of the right kidney in the portal venous phase. Data were transferred to the General Electric Advantage Workstation 4.4 for further data analysis.

### Gross tumor volume measurement

**Figure 1:**
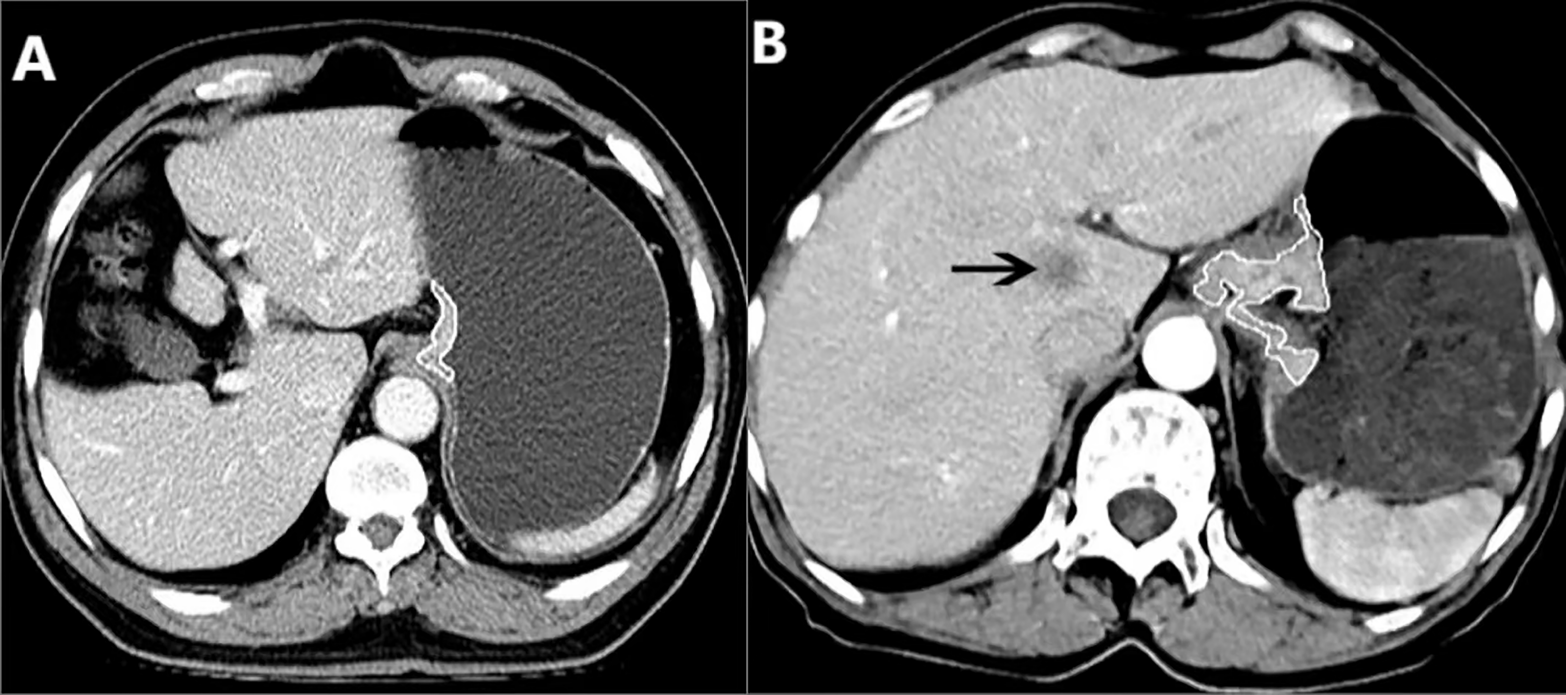
**(A)** In a 71-year-old male with resectable adenocarcinoma of the esophagogastric junction staged as cT_3_N_1_M_0_, preoperative enhanced CT shows the total tumor volume obtained by manual sketching layer by layer along the edge of abnormal distal esophageal and proximal gastric wall, and the total tumor volume is 10.20 cm^3^. **(B)** In a 70-year-old female with unresectable adenocarcinoma of the esophagogastric junction staged as cT_3_N_1_M_1_, the enhanced CT shows the metastasis in the liver (arrow), and the total volume of the tumor is measured by manual sketching layer by layer along the edge of the thickened distal esophageal and proximal gastric wall, and the total volume of the tumor is 35.62 cm^3^.

The GTV of AEG was measured on the above mentioned workstation, was calculated by multiplying the sum of all the tumor areas by the section thickness according to the method used in previous reports (10, 16). For the measurement of tumor area on transverse images, the thickness of distal esophageal and gastric wall greater than 5 mm during the gastric dilation was defined as abnormal (17, 18). Subsequently, the shape of the AEG was manually delineated along the edge of the thickened distal esophageal and proximal gastric walls (**Figure 1**) on each contiguous tumor section in the portal venous phase image for the reason that the contour of the tumor can be better shown in the portal venous phase than in the arterial phase. Finally, the tumor areas were added and then multiplied by the layer thickness to obtain the GTV. It took less than 5 minutes to outline the primary tumor to obtain GTV for each patient. To minimize the GTV measurement errors, care was taken to avoid air and liquid within the esophageal and/or gastric lumen. To reduce the measurement bias, the GTV was measured independently by two experienced radiologists (Observer 1 and 2, each with 3 years of radiology expertise), who were blinded to the surgical outcome and clinical details at the time of delineation. Before the previous radiologists delineated the tumor to obtain the GTV, a professor of radiology with 24 years of experience in radiology diagnosis trained them how to draw the tumor contour in 10 patients at random. To verify the intraobserver reproducibility, the measurement of all the tumors were repeated by Observer 1 one month later.

### Statistical analysis

All the statistical analysis of data were performed by using software (version 26.0 for Windows; SPSS, Chicago, IL, USA). A test with *P* value less than 0.05 was defined as statistically significant. The inter- and intraobserver intraclass correlation coefficient (ICC) was used to assess the reliability of measurements of GTV. ICCs less than 0.5, between 0.5 and 0.75, between 0.75 and 0.9, and greater than 0.90 are indicative of poor, moderate, good and excellent reliability, respectively (19).

The continuous variables were expressed as mean ± standard deviation (SD). Categorical variables were shown as numbers and percentages. For TC, the univariate association of GTV and clinical factors including age, sex, Siewert classification, cT, cN stages and vascular invasion were assessed by using χ^2^ test. If the variables with a *P* value less than 0.05 were regarded statistically different, they were included in the multivariate analysis, which was carried out through the binary logistic regression analysis to clarify the independent determinants of resectability of AEG. The Mann-Whitney U test was used to compare GTV corresponding to different cT and cN stages between patients with resectable and unresectable tumors. When the previous Mann-Whitney U test showed a significant difference, receiver operating characteristic (ROC) analyses were then carried out to determine whether the cutoff values of GTV corresponding to cT and cN stages could be helpful to determine resectability (9). In VC, Cohen’s Kappa tests were used to evaluate performances of the previous ROC models to independently determine resectability according to the following rating scheme: less than 0.20, between 0.21 and 0.40, between 0.41 and 0.60, between 0.61 and 0.80, and greater than 0.81 are indicative consistency of slight, fair, moderate, substantial and almost perfect, respectively (20).

## Results

### Intra- and interobserver reproducibility of GTV measurements in TC

In all the 279 patients with AEG in TC, the initial measured mean GTV of the first observer was 36.58 ± 26.61 cm^3^. The intra- and interobserver ICC values of GTV measurement were 0.996 (95% confidence interval [95%CI], 0.995–0.997) and 0.998 (95%CI, 0.998–0.999), respectively (both *P*-values < 0.0001), indicating that measurement of GTV obtained excellent repeatability in TC. Therefore, the first measurement of the first observer was repeatable and could be used for further statistical analysis.

### Univariate analysis: Correlation of clinical factors and GTV with resectability in TC

The clinical factors and GTV together with their correlations with resectability are summarized in **Table 3**. The Siewert classification, cT and cN stages, vascular invasion, and GTV were related to the resectability. Specifically, when primary tumor and/or metastatic lymph nodes invaded the adjacent vessels, indicating that the tumor could be removed less likely. Siewert III AEG is less likely to be resected than Siewert I/II, patients with higher cT stage were less likely to be treated surgically, and patients with higher cN stage were associated with lower possibility of resection (all *P*-values < 0.05).

**Table 3:** Univariate analysis of clinical factors and gross tumor volume correlated with resectability in the training cohort.

Variable	Resectable (n = 184)	Unresectable (n = 95)	*P*-value
Sex			0.818
Male	136 (73.9)	69 (72.6)	
Female	48 (26.1)	26 (27.4)	
Age			0.720
<67	83 (45.1)	45 (47.4)	
>67	101 (54.9)	50 (52.6)	
Siewert classification			0.011
I	3 (1.6)	1 (1.1)	
II	129 (70.1)	50 (52.6)	
III	52 (28.3)	44 (46.3)	
T stage			<0.0001
cT_1_	8 (4.4)	0	
cT_2_	23 (12.5)	0	
cT_3_	86 (46.7)	16 (16.8)	
cT_4a_	67 (36.4)	73 (76.8)	
cT_4b_	0	6 (6.4)	
N stage			<0.0001
cN_0_	35 (19.0)	0	
cN_1_	56 (30.5)	7 (7.4)	
cN_2_	81 (44.0)	30 (31.6)	
cN_3_	12 (6.5)	58 (61.0)	
Vascular invasion			<0.0001
Yes	36 (19.6)	61 (64.2)	
No	148 (80.4)	34 (35.8)	
Gross tumor volume (cm^3^)			
<36.58	149 (81.0)	23 (24.2)	<0.0001
>36.58	35 (19.0)	72 (75.8)	

The numbers in the parentheses are percentages.

### Multivariate analysis: Independent determinants of resectability in TC

According to the above significant factors obtained by the univariate analysis, Siewert classification, vascular invasion, cT stage, cN stage and GTV were chosen as potential independent determinants of resectability, and the binary logistic regression analysis was carried out to determine the independent factors. Logistic regression analysis indicated that GTV, cT and cN stages were independent determinants between resectable and unresectable AEG (all *P-*values <0.0001, odds ratio = 4.715, 4.534 and 1.107, 95%CI of 2.016–11.026, 2.403–8.557 and 1.070–1.145, respectively).

### GTV corresponding to cT and cN stages: Resectable vs. unresectable AEG in TC

Mann-Whitney U test was used to analyze differences in GTV corresponding to cT and cN stages between resectable and unresectable AEG. Because numbers of patients with tumors in stage cT_1_-_2_ and cN_0_ were small, and all tumors in stage cT_1_-_2_ and cN_0_ were defined as resectable tumors in our study based on the NCCN guidelines, we did not conduct the statistical tests on the previous populations between resectable and unresectable lesions. Since most of cases in our study were staged as cT_3_ and cT_4_ category, we performed the Mann-Whitney U tests in the populations with resectable vs. unresectable tumors in stages cT_3_ and cT_4_, or cN_1_, cN_2_, and cN_3_. As described in the statistical tests, GTV could be different between patients with resectable and unresectable tumors in stages cT_1-4_N_0-3_, especially in stage cT_3_ and cT_4_, or in stage cN_1_, cN_2_, and cN_3_ (all *P*-values < 0.01).

### ROC analyses of GTV corresponding to cT and cN stages to distinguish between resectable and unresectable tumors

**Figure 2:**
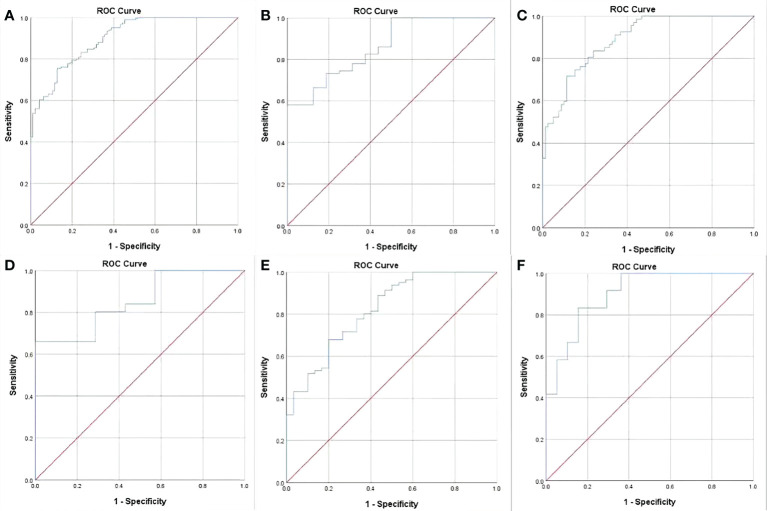
Receiver operating characteristic (ROC) analysis of gross tumor volume (GTV) of adenocarcinoma of the esophagogastric junction has been performed for determining resectability, and the ROC curves show that the GTV can help identify whether the tumor can be resectable in stages cT_1-4_N_0-3_ **(A)** with the cutoff values of 32.77 cm^3^, especially in stages cT_3_ **(B)** and cT_4_ **(C)**, or stages cN_1_ **(D)**, cN_2_ **(E)** and cN_3_ **(F)** with the threshold values of 27.67 and 32.77 cm^3^, or 27.09, 33.32 and 37.39 cm^3^, respectively.

In order to assess GTV corresponding to cT and cN stages in determining the resectability of AEG, the ROC analysis was carried out. According to the ROC analysis (**Figure 2**), the GTV could be helpful to determine resectability of AEG in stages cT_1-4_N_0-3_ with the cutoff value of 32.77 cm^3^, especially in stages cT_3_ and cT_4_, or stages cN_1_, cN_2_ and cN_3_ with the cutoff values of 27.67 and 32.77, or 27.09, 33.32 and 37.39 cm^3^, respectively. The area under the ROC curve (AUC), specificity, sensitivity, predictive value and accuracy of GTV corresponding to cT and cN stages for determining resectability of AEG in TC and VC are shown in **Table 4**.

**Table 4:** Receiver operating characteristic analysis of gross tumor volume corresponding to cT and cN stages for determining resectability of adenocarcinoma of the esophagogastric junction in the training and validation cohorts.

Category	Cutoff (cm^3^)	AUC	Sensitivity (%)	Specificity(%)	PPV (%)	NPV (%)	Accuracy (%)
The training cohort							
cT_1-4_N_0-3_	32.77	0.901	75.5	87.4	92.0	0.648	0.796
T Stage							
cT_3_	27.67	0.860	58.1	100.0	100.0	0.308	0.647
cT_4_	32.77	0.890	71.6	88.6	84.2	0.787	0.808
N Stage							
cN_1_	27.09	0.852	66.1	100	100	0.269	0.698
cN_2_	33.32	0.821	67.9	80.0	90.2	0.480	0.712
cN_3_	37.39	0.902	83.3	84.5	52.6	0.961	0.843
The validation cohort							
cT_1-4_N_0-3_	30.98	0.828	73.0	85.2	87.1	69.7	78.1
T Stage							
cT_3_	31.27	0.744	77.8	80.0	93.3	50.0	78.3
cT_4_	32.99	0.756	61.5	83.3	66.7	80.0	75.7
N Stage							
cN_1_	31.11	0.786	85.7	66.7	92.3	50.0	82.4
cN_2_	33.33	0.821	62.5	85.7	90.0	50.0	69.6
cN_3_	35.31	0.824	23.5	76.5	33.3	100.0	78.9

AUC, area under the receiver operating characteristic curve; PPV, positive predictive value; and NPV, negative predictive value.

### Cohen’s kappa tests for verifying performance of the ROC models in VC

In order to verify the performance of our ROC models of GTV in stage cT_1-4_N_0-3_, especially in stages cT_3_ and cT_4_, or stages cN_1_, cN_2_ and cN_3_ to distinguish between resectable and unresectable AEG lesions in TC, Cohen’s Kappa tests were performed in VC according to the obtained cutoff values. The tests revealed that the models obtained good agreements in VC as shown in **Table 5**.

**Table 5:** Cohen’s kappa tests in the validation cohort for verifying the performance of our receiver operating characteristic models.

Category	Cohen K value	95%CI	*P*-value
cT_1-4_N_0-3_	0.938	0.842-1.000	<0.0001
T stage			
cT_3_	0.907	0.657-1.000	<0.0001
cT_4_	1.000	1.000-1.000	<0.0001
N stage			
cN_1_	0.721	0.261-1.000	0.002
cN_2_	1.000	1.000-1.000	<0.0001
cN_3_	0.776	0.410-1.000	0.001

95%CI, 95% confidence interval.

## Discussion

Our study explored the potential determinants associated with the resectability of AEG including the clinical factors and GTV, and developed the combined ROC models to quantitatively identify the resectability. The current research showed that the cT stage, cN stage, Siewert classification, vascular invasion and GTV could be related to the resectability of AEG according to the univariate analysis. Based on our multivariate analysis, cT stage, cN stage and GTV were independently associated with the resectability. Considering the independent factors, we further demonstrated that GTV corresponding to cT and cN stages could help quantitatively determine the resectability.

As shown in our study, the GTV of AEG measured on CT could be independently associated with the resectability. Gross tumor volume can be used as a comprehensive index to reflect the tumor diameter, length and depth of invasion. Previous study has shown that measuring the gross tumor volume of AEG by CT can help determine lymph node metastasis (10). For AEG, PET/CT measurement of GTV can help determine the systemic spread of the tumor, suggesting that gross tumor volume might play an important role in the staging of the tumor to determine whether it can be resectable, so as to help select the appropriate treatment scheme (5, 21). Therefore, we can speculate that the larger GTV of AEG were associated with the lower possibility of resection.

Our study demonstrated that cT stage could be independently associated with the resectability of AEG. A published study has shown that the exact cT stage could be very crucial for the limited resection of AEG in early period, and it was also important to exclude patients with advanced diseases from unnecessary surgery (22). We can presume that cT stage of AEG could affect the choice of treatment (12, 13). We can speculate patients with AEG in higher cT stage could be less likely to be treated surgically.

Another independent determinant of resectability of AEG was the cN stage in our study. Studies show that lymph node status affects the prognosis of patients with AEG, often complicated with vascular and nerve invasion (23, 24). It has been reported that accurate judgment of lymph node metastasis and N stage is extremely important to determine the treatment mode of AEG (16). Based on the above researches, we can speculate patients with more lymph nodes metastasis could be associated with lower possibility of resection.

As shown in our research, Siewert classification and vascular invasion could be potential independent determinants of resectability for AEG. However, our study revealed that the Siewert classification and vascular invasion of AEG were not independently associated with the resectability in the multivariate analysis. We usually determine the Siewert classification of AEG according to the location of the center of the tumor (3). Burkhard et al. reported that the appropriate surgical procedure was different due to the different Siewert classification of AEG (25). We can presume that Siewert classification may be related to surgical procedure of AEG, but there is no relationship with the resectability. According to NCCN guidelines, we knew that the AEG with adjacent vascular invasion was defined unresectable. Some studies showed that vascular invasion could be an independent influencing factor of lymph node metastasis, which had a great relationship with the progress of the tumor (23, 26). But our multivariate analysis showed that the vascular invasion by the primary tumor (cT_4b_) or by metastatic lymph nodes (generally appearing as multi-station and bulky lymphadenopathy) could have no relationship with resectability, which may be explained by that the tumor with the higher cT stage and/or cN stage have yet been independent factors of resectability.

Our study suggested that cT stage, cN stage and GTV of AEG may be independently associated with the resectability. Therefore, we compared GTV between resectable and unresectable tumors in different cT and cN stages, especially in stages cT_3_ and cT_4_, or cN_1_, cN_2_ and cN_3_. Our study showed that the stratification of GTV according to cT and cN stages had a good decisive performance for determining the resectability. Corresponding to cT or cN stages, especially to stages cT_3_ and cT_4_, or to stages cN_1_, cN_2_ and cN_3_, the AUC values to determine resectability could be greater than 0.80. Moreover, the previous ROC models obtained good performance in distinguishing resectable and unresectable AEG with Cohen’s K values of greater than 0.71 in stage cT_1-4_N_0-3_ in VC, especially in stages cT_3_ and cT_4_, or N_1_, N_2_ and N_3_.

In general, we developed a quantitative method to determine whether AEG could be resectable according to GTV in consideration of other independent determinants including cT or cN stages. Nonetheless, AEG of Siewert type III with lymph node infiltration at the root of the mesentery or para-aorta, and AEG of Siewert I/II with multi-station bulky lymph node metastasis should be considered unresectable demonstrably on CT. The patients with definite distant metastases could also be considered unresectable. For the patients with locally advanced tumors in the absence of distant metastases and multi-station lymph node metastases especially in stage T_4_, however, our ROC quantitative method could be more effective in determining whether AEG can be resectable.

There were several limitations in our study. The first limitation was the nature of single-center retrospective study. But the AUC values of our ROC models to determine resectability of AEG were greater than 0.8, indicating good performance in distinguishing between resectable and unresectable AEG. We will carry out a prospective multi-center study to confirm our results. Secondly, the measurement of GTV might be affected by the distention of the esophagus and stomach. In order to reduce the error affected by the expansion of esophagus and stomach during obtaining GTV, we measured the tumor volume by manual sketching the edge of the tumor independently by two experienced radiologists, and obtained excellent repeatability. Thirdly, some factors such as tumor biomarkers may affect the resectability of AEG. We will perform the relevant study focusing on the combination of imaging and laboratory data to improve our ROC models for determining the resectability in the future. Fourthly, it may be more complicated and time-consuming to obtain GTV than TNM stage to determine the resectability. Despite this limitation, we reported independent determinants to develop ROC models to quantitatively determine the resectability.

In conclusion, we found that GTV, cT and cN stages could be independently associated with the resectability of AEG. And the GTV measured on CT corresponding to cT and cN stages may be helpful to determine resectability as shown by our ROC analyses. We hope that this study may help quantitatively determine whether AEG can be surgically removed, so that clinicians can choose the best treatment for individual cases.

## Data availability statement

The raw data supporting the conclusions of this article will be made available by the authors, without undue reservation.

## Ethics statement

The studies involving human participants were reviewed and approved by the institutional ethics committee of Affiliated Hospital of North Sichuan Medical College. The patients/participants provided their written informed consent to participate in this study.

## Author contributions

T-WC, X-MZ, RL, and H-YZ: study conception and design. K-YL, JO, Z-YY, DG, and X-YY: data collection and analysis. K-YL, DG, and Z-YY: image processing and modeling. K-YL: manuscript writing. K-YL, JO, and X-YY: statistical analysis. All authors contributed to the article and approved the submitted version.

## Conflict of interest

The authors declare that the research was conducted in the absence of any commercial or financial relationships that could be construed as a potential conflict of interest.

## Publisher’s note

All claims expressed in this article are solely those of the authors and do not necessarily represent those of their affiliated organizations, or those of the publisher, the editors and the reviewers. Any product that may be evaluated in this article, or claim that may be made by its manufacturer, is not guaranteed or endorsed by the publisher.

## Abbreviations

AEG, adenocarcinoma of the esophagogastric junction; AUC, area under the receiver operating characteristic curve; CI, confidence interval; CT, computed tomography; GTV, gross tumor volume; ICC, intraclass correlation coefficient; NCCN Guidelines, National Comprehensive Cancer Network Clinical Practice guidelines; ROC, receiver operating characteristic; TC, training cohort; VC, validation cohort.
